# Surviving the Odds: Primary Cardiac Lymphoma With Locoregional Lymph Node Involvement Complicated by Pericardial Effusion and Arrhythmia

**DOI:** 10.7759/cureus.81012

**Published:** 2025-03-22

**Authors:** Kinan Bachour, Sagar Shah, Allison Reichl, Jennifer Chia, David S Shabsovich, Adrian Castillo, Geoffrey Cho

**Affiliations:** 1 Department of Cardiology, University of California Los Angeles, Los Angeles, USA; 2 Department of Gastroenterology, University of California Los Angeles, Los Angeles, USA; 3 Department of Internal Medicine, University of California Los Angeles, Los Angeles, USA; 4 Department of Pathology and Laboratory Medicine, University of California Los Angeles, Los Angeles, USA

**Keywords:** bradycardia, cardiac lymphoma, cardio-oncology, case report, electrophysiology

## Abstract

Primary cardiac lymphoma (PCL) is a rare malignant disease and its presentation varies depending on the degree of infiltration and location in the myocardium; hence its diagnosis can be challenging. We present a case of a 67-year-old otherwise healthy male who initially presented with recurrent syncope found to have a pericardial effusion and complete heart block. Paratracheal biopsy of a fluorodeoxyglucose (FDG)-avid lymph node was consistent with diffuse large B-cell lymphoma. Diagnosis is usually identified histologically, but imaging is key in guiding biopsy and monitoring disease regression. Chemotherapy is the mainstay of treatment. In this case report, we present a unique presentation of primary cardiac lymphoma presenting as complete heart block with extra-cardiac lymph node involvement and review its management.

## Introduction

Primary cardiac lymphoma (PCL) is a rare malignant disease that accounts for around 1.3% of all cardiac tumors, which compared to other forms of malignancies, are already rare in and of themselves, representing only 0.02% of all tumors [1,2]. While there is no single unifying criteria to diagnose PCL, PCL is generally defined by involvement of the pericardium and myocardium and/or cardiac involvement without evidence of lymphoma in other organs [3,4]. Although rare, the most common histological type of PCL is large B cell lymphoma. PCL is usually fatal, diagnosed on autopsy, which makes early diagnosis critical as chemotherapy is the mainstay of treatment [5]. We present a unique case of diffuse large B cell cardiac lymphoma with involvement of regional lymph nodes causing conduction abnormalities leading to complete heart block and syncope. This case was previously presented at the 2023 annual American College of Cardiology Meeting.

## Case presentation

A 67-year-old male with a history of gout and gastroesophageal reflux disease presented with recurrent syncopal episodes. The patient reported experiencing a syncopal episode after having a bowel movement and standing up from the toilet. The syncopal episode was witnessed; his partner reported that the patient grew faint, lost consciousness as he fell to the floor, and spontaneously regained consciousness after 10-15 seconds. There was no report of seizure-like activity. The patient reported that he had experienced between 3-5 syncopal events over the past three years - predominantly in the setting of defecation but also during a bout of laughter, thought to be vasovagal. The patient denied nausea, vomiting, fevers, chills, weight loss, or recent travel history. The patient did not smoke tobacco or drink alcohol. There was no family history of sarcoid or infiltrative diseases.

On physical examination, the patient appeared euvolemic. There were no cardiac murmurs, rubs or gallops appreciated, though heart sounds were slightly soft/diminished. Lungs were clear to auscultation bilaterally. No motor or sensory neurologic deficits. There was no cervical, supraclavicular, submandibular or submental lymphadenopathy. Orthostatic vitals were negative, and the patient was noted to remain in asymptomatic bradycardia throughout.

**Figure 1:**
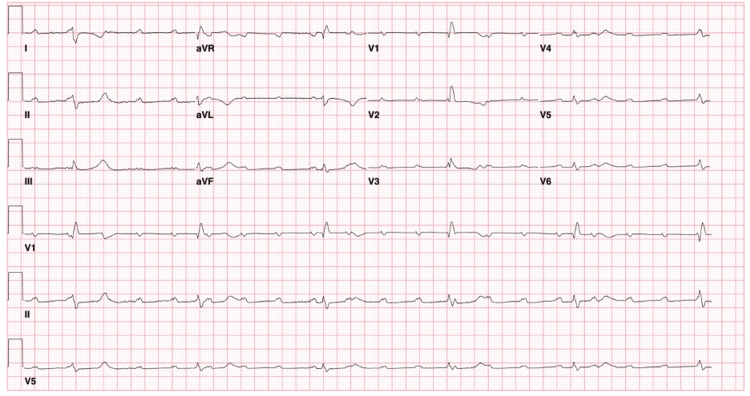
EKG on admission Sinus tachycardia with complete heart block with likely infra-Hisian junctional escape beat (QRS 136). Notably, electrocardiogram has low voltage likely secondary to pericardial effusion, infiltrative process of myocardium walls (refer to Figure 2) and body habitus.

**Figure 2:**
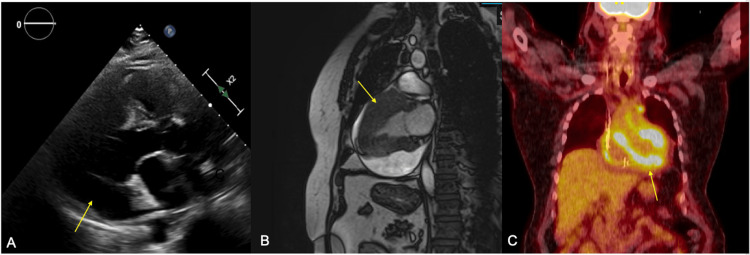
Imaging results (A) Echocardiogram demonstrating a moderate posterior pericardial effusion (free space of ~17 mm) marked with an arrow. Ejection Fraction of 60-65%, visually estimated with moderate concentric left ventricular hypertrophy (interventricular septal diameter of 1.4 cm), indeterminate diastolic function (Left atrial volume index of 19 ml/m^2^, Tricuspid regurgitant jet 2.1 m/s, E/e’ ratio 27, lateral e’ 4.5 and medial e’ 4.7). (B) Cardiac MRI demonstrating 20 mm thickness in the anterior septum, 30 mm mid-anterior wall (marked with an arrow), and inferior wall of 11 mm. Right ventricular walls also thickened. (C) PET CT scan with regional FDG uptake in the more basal portions of the anterior, inferior and lateral walls of the left ventricle, consistent with infiltrative cardiomyopathy, myocarditis or an inflammatory process. There was also para-aortic lymph node uptake.

On further investigation, the complete blood count, metabolic panel, and thyroid panel all returned within normal limits. Troponin I had an acute rise and fall with a peak at 2.21 ng/mL (Table 1). Electrocardiogram (ECG) was significant for HR~40-50s beats per minute, complete heart block, low voltage, and absence of signs of ischemia (Figure 1). Echocardiogram (Figure 2A) showed moderate concentric left ventricular hypertrophy, normal systolic function, indeterminate diastolic function, mildly enlarged right ventricular size and reduced systolic function. Moderate posterior pericardial effusion was present without tamponade. Cardiac magnetic resonance imaging (cMRI) (Figure 2B) demonstrated 20 mm thickness in the anterior septum, 30 mm mid-anterior wall, and inferior wall of 11 mm. The right ventricular wall was also thickened. The infiltrative process was noted to have a patchy appearance of late-gadolinium enhancement.

**Table 1:** Pertinent Laboratory Values ESR: Erythrocyte Sedimentation Rate; BNP: B-type Natriuretic Peptide; ACE: Angiotensin-Converting Enzyme; CRP: C-Reactive Protein; WBC: White Blood Count

Lab	Value	Reference
Troponin I peak	2.21 ng/ml	<0.04 ng/ml
ESR	14 mm/hr	<12 mm/hr
BNP	364 pg/ml	<100 pg/ml
ACE	12 U/L	16-85 U/L
CRP	0.4 mg/dl	<0.8 mg/dl
Serum Kappa Light Chains	16 mg/L	3.3-19.4 mg/L
Serum Lambda Light Chains	12 mg/mL	5.7-26.3 mg/L
Kappa/Lambda Ratio	1.33	0.26-1.65
WBC	11.7x10^3^/ul	4.1-10.0x10^3^/ul
Neutrophil %	84.0%	-
Lymphocyte %	10.0%	-
Monocyte %	5.1%	-
Eosinophil	0.1%	-
Basophil	0.2%	-

Left heart catheterization showed single vessel obstructive coronary artery disease with a 60-70% tapering proximal left anterior descending (LAD) lesion. Cardiac positron emission tomography cardiac scan (PET) (Figure 2C) exhibited intense regional fluorodeoxyglucose (FDG) uptake in the more basal portions of the anterior, inferior and lateral walls of the left ventricle, consistent with possible infiltrative cardiomyopathy, myocarditis or an inflammatory process. Para-aortic and paratracheal lymphadenopathy with intense FDG uptake was also exhibited. Standard laboratory markers were negative. The patient also had normal angiotensin-converting enzyme levels (usually elevated in 60-80% of patients with sarcoidosis), serum and urine electrophoresis, immunofixation, ESR, CRP, and no eosinophilia (Table 1). At that point differential diagnosis narrowed towards possible malignancy causing complete heart block secondary to myocarditis or tumor infiltration.

**Figure 3:**
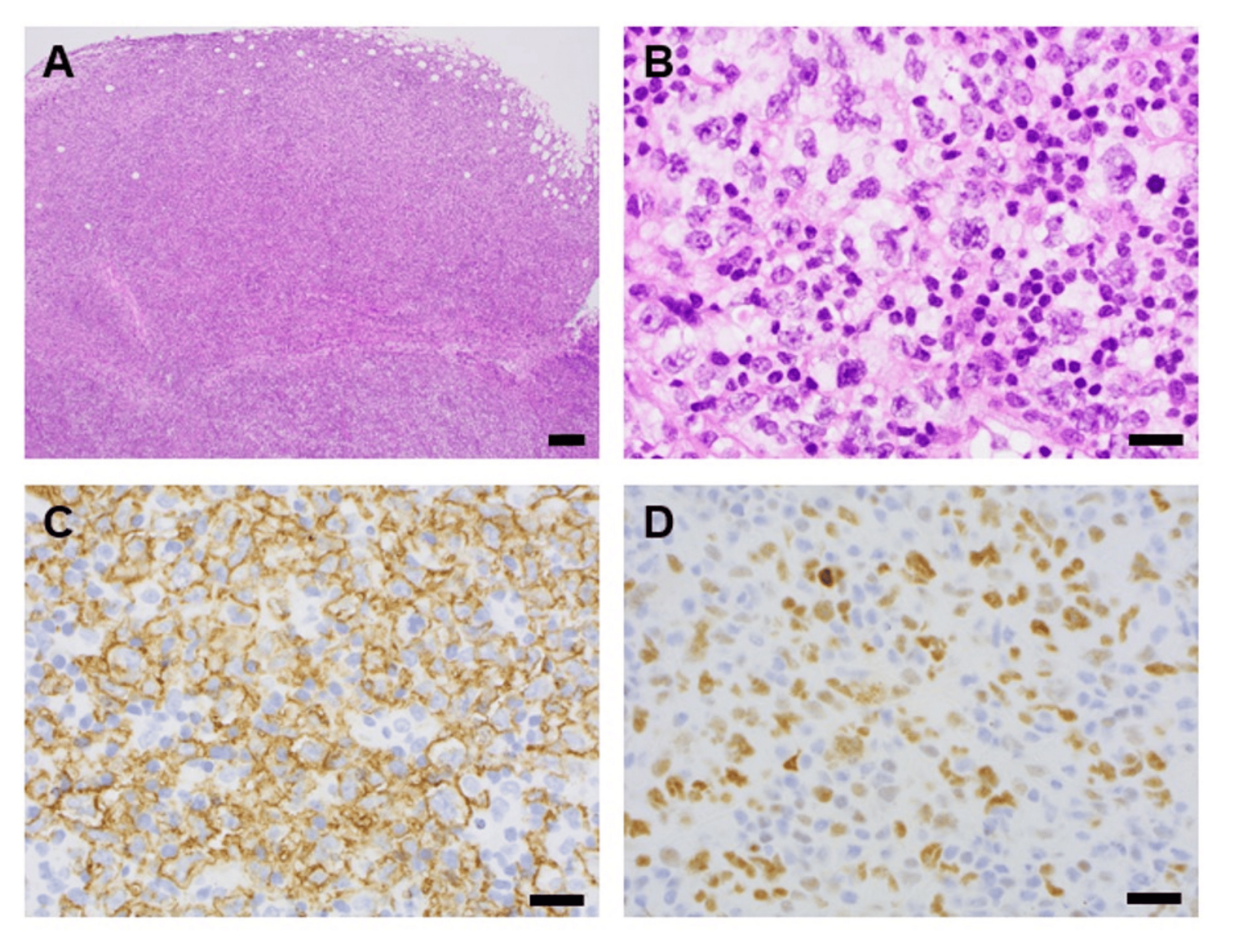
Pathologic findings of paratracheal lymph node biopsy Tissue was fixed in formalin and processed for histologic sectioning, with hematoxylin & eosin (H&E) stained sections showed diffuse architectural effacement (A) by sheets of large neoplastic lymphocytes (B). Immunohistochemical staining (IHC, brown chromogen, with hematoxylin nuclear counterstain) was positive for CD20 (C) and cMYC (D). Scale bars represent 200 um (A) or 20 um (B-D).

Initially, the patient's initial junctional escape rhythm was stable but then degenerated during hospitalization. The patient was noted to develop a slower rate of ~30 bpm with QRS widening (right bundle branch block + left posterior fascicular block) concerning for progression of conduction disease. Urgent temporary-permanent pacemaker was placed. Cardiothoracic surgery was consulted and performed a diagnostic thoracic lymphadenectomy and cervical mediastinoscopy. Pathology findings demonstrated diffuse large B-cell lymphoma (DLBCL), germinal center B-cell phenotype (Figure 3). Immunostaining highlighted diffuse sheets of large neoplastic lymphocytes positive for PAX5, CD19, CD20, CD10 (weak, focal), CD23, CD30, BCL2 (weak), BCL6 and MYC with estimated Ki67 proliferation index 70%, and negative for CD3, BCL1, MUM1, and EBV-EBER by in-situ hybridization (Table 2). FISH studies detected loss of one copy of the 5’BCL6-specific signal, suggestive of 5’BCL6 deletion or unbalanced BCL6 rearrangement; such findings have been associated with an aggressive clinical course in DLBCL [6]. No gene rearrangements involving BCL2 or cMYC were detected.

**Table 2:** Immunostains of Lymph Node Tissue PAX5: Paired Box 5; CD: Cluster of Differentiation; BCL: B-Cell Lymphoma; MYC: Cellular Myelocytomatosis Oncogene (c-Myc); MUM1: Multiple Myeloma Oncogene 1; EBV-EBER (in-situ hybridization): Epstein-Barr Virus–Encoded RNA (EBER) in-situ hybridization

Stains	Findings	Reference Value
PAX5	Positive (B-cell marker)	Positive (B cells)
CD19	Positive (B-cell marker)	Positive (B cells)
CD20	Positive (B-cell marker)	Positive (B cells)
CD10	Weak, focal positive	Positive or negative
CD23	Positive	Positive or Negative
CD30	Positive	Negative (unless activated B/T cells)
BCL2	Weak positive	Positive or Negative
BCL6	Positive (Germinal center marker)	Positive or Negative
MYC	Positive	Negative (unless aggressive lymphoma)
Ki-67	~70% (high proliferation)	<40% in reactive lymphoid tissue
CD3	Negative (T-cell marker)	Positive (T cells)
BCL1 (Cyclin D1)	Negative	Negative (unless mantle cell lymphoma)
MUM1	Negative	Positive or Negative
EBV-EBER (in-situ hybridization)	Negative	Negative (unless EBV-associated lymphoma)

Oncology was consulted and planned to initiate both intravenous and intrathecal chemotherapy regimen involving rituximab, etoposide, prednisone, vincristine, cyclophosphamide and doxorubicin (R-EPOCH) during the admission. Intrathecal chemotherapy was thought to be critical due to the risk of central nervous system involvement in aggressive B-cell lymphomas. Prior to chemotherapy, given his cardiac syncope likely due to ventricular arrhythmias and the high probability of arrhythmias with chemotherapy and intracardiac chemo-mediated cell lysis, a dual chamber implantable cardioverter defibrillator (ICD) device was placed. The patient tolerated the first inpatient cycle of chemotherapy well without any evidence of sustained ventricular tachycardia with treatment. He was discharged with outpatient follow-up for continued chemotherapy.

## Discussion

The differential diagnosis of syncope is broad and generally includes cardiac, neuro, orthostatic and vagal/reflex etiologies. While seizures and strokes were also included in the differential, the patient’s lack of post-ictal state, absence of seizure-like activity during the event, and non-focal neurologic exam made these less likely. The occurrence of syncopal episodes in the setting of bowel movements and laughter both suggested initially a preload-dependent mechanism, given increased intraabdominal pressure or vagally mediated but could have been arrhythmogenic.

His cardiac MRI revealed a possible infiltrative process that was suspicious of either sarcoidosis, amyloidosis, or hemochromatosis. Given the absence of other clinical signs of infiltrative process and PET CT findings with lymph node penetration, oncology was consulted for suspicion of malignancy. While the diagnosis of cardiac lymphoma would have been compatible with the patient’s rapidly progressing conduction abnormalities, the specific pattern of lymph node involvement is much less typical of PCL. PCL usually presents in a single region of the heart, classically right-sided, but in our patient, it was more diffusely distributed throughout the heart [7]. The team considered diagnostic studies including peripheral blood and pericardial fluid flow cytometry, but these methods were deemed likely to be lower yield than surgical biopsy [8]. A biopsy of the paratracheal lymph node was then performed. Notably, our diagnosis of likely cardiac involvement of B-cell lymphoma was made based on lymph node biopsy, saving the patient from potentially higher-risk cardiac biopsy histological examinations.

There are case reports of ventricular arrhythmias with lymphoma involving the heart [9,10]. Given that the intended treatment modality was chemotherapy with directed cell lysis, the patient was at risk for ventricular arrhythmias and the patient and team decided to proceed with dual chamber ICD in addition to his pacemaker indication given the high myocardial burden. Despite PCL having a high fatality rate, currently, our patient is fortunately doing well with improvement in cardiac involvement on chemotherapy with occasional return of native AV conduction. No sustained ventricular arrhythmias were noted during treatment. Cardiac MRI after treatment was notable for the decrease in pericardial effusion size, with persistent delayed enhancement of similar myocardial regions but with significant improvement from initial imaging. No adverse events or complaints were noted at the time of his outpatient follow-up.

## Conclusions

Primary cardiac lymphoma is a rare malignant disease with a poor prognosis. Diagnosis is challenging as presentation is nonspecific, though early diagnosis and treatment is critical for prolonging survival. PCL can present with arrhythmias leading to syncope. Diagnosis is usually identified histologically and chemotherapy is the mainstay of treatment. Our patient who presented with syncope and complete heart block was found to have PCL diffusely in the heart causing conduction disease with locoregional lymph node involvement complicated by pericardial effusion. Despite the poor prognosis of PCL, fortunately, at the time of publication, the patient has tolerated two cycles of R-EPOCH and is now doing well with improvement in cardiac function, conduction disease and pericardial effusion size. This highlights the importance of early diagnosis for improving prognosis.
